# Epidemiological investigation of non-carious cervical lesions and possible etiological factors

**DOI:** 10.4317/jced.54860

**Published:** 2018-07-01

**Authors:** Veljko Kolak, Dragana Pešić, Irena Melih, Marija Lalović, Ana Nikitović, Ankica Jakovljević

**Affiliations:** 1 Assistant Professor, DDS, PhD, Department of Restorative Dentistry and Endodontics, Faculty of Dentistry in Pančevo, Serbia; 2 Teaching Assistant, DDS, Department of Restorative Dentistry and Endodontics, Faculty of Dentistry in Pančevo, Serbia; 3 Professor, DDS, PhD, Department of Restorative Dentistry and Endodontics, Faculty of Dentistry in Pančevo, Serbia

## Abstract

**Background:**

Epidemiological studies of non-carious cervical lesions (NCCLs) are being conducted in all geographical regions, which is completely justified, considering the high frequency of these lesions and possible consequences. Data obtained from such studies are of great importance because, beside describing the extent and degree of lesions, they can also point to specific etiological factors. The purpose of this study was to analyze the frequency and distribution of NCCLs among the patients of Faculty of Dentistry in Pancevo, Serbia, and to investigate the impact of certain etiological factors on the frequency of NCCLs.

**Material and Methods:**

The study included 394 patients, who were clinically examined for the presence of NCCLs and interviewed about potential etiological factors using specially designed questionnaire. Saliva samples were analyzed for 30 patients with multiple NCCLs (≥3) and 30 patients without signs of cervical lesions and restorations. Subject – level logistic regression was used to analyze the association of potential etiological factors and presence of NCCLs and Wilcoxon test for the quantity and quality of saliva.

**Results:**

NCCLs were diagnosed at 68.5% from total number of respondents, 15% from all present teeth were affected. The highest prevalence was recorded on premolars. Presence of lesions significantly increased with age. Frequent consumption of citrus fruit was associated with the presence of NCCLs. Significantly lower frequency of NCCLs was recorded among subjects who frequently chew gums. Significantly lower pH values of unstimulated and stimulated saliva were recorded in the group of patients with multiple NCCLs compared to control group.

**Conclusions:**

This study showed high frequency of NCCLs among subjects of different age. Premolars were the most frequently affected. Age, frequent consumption of citrus fruit and lower salivary pH value were associated with an increased occurrence of NCCLs. Chewing gums habit was associated with an decreased occurrence of NCCLs.

** Key words:**NCCL, abrasion, erosion, abfraction, saliva.

## Introduction

Non-carious cervical lesion (NCCL) can be defined as the loss of tooth structure at the cemento-enamel junction that is unrelated to dental caries (1). The neck of the tooth morphology and histology differs from the crown and root. Enamel gradually becomes thinner coming close to enamel-cement junction, and that is the reason why cervical region represents the most vulnerable place, where dentin is likely to be exposed to the action of irritant agents. The enamel prisms direction changes into a flattened one, in contrast with their undulating direction in crown enamel portion. Due to the flat surface of enamel-dentin junction, the mechanical interlocking between enamel and dentin in the cervical area is weaker than that in the other regions with a serrated appearance. Also, the cervical region of the tooth is the region of aprismatic enamel which contains less mineral and is physically thinner than the rest of prismatic enamel (2). Numerous theories about the formation of NCCLs centers on abrasive damage caused primarily by tooth brushing, and erosion caused by acid of a non-bacterial origin, which may be either intrinsic or extrinsic. In the past 30 years, it was hypothesized that the etiological factor of these wedge-shaped defects is tooth flexure resulting from tensile stress. Different terminologies in the literature, such as “cervical erosion”, “cervical abrasion” and “abfraction” essentially describe similar lesions.

Epidemiological studies of NCCLs are being conducted in all geographical regions, which is completely justified, considering the high frequency of these lesions and possible consequences. On the other hand, data obtained from such studies are of great importance because, beside describing the extent and degree of lesions, they can also point to specific etiological factors.

The purpose of this study was to analyze the frequency of non-carious cervical lesions among the population of patients who referred to Faculty of Dentistry in Pancevo, Serbia, to analyze the distribution of lesions by gender and age, tooth functional group, jaw, arch side and tooth surface, as well as to investigate the impact of certain etiological factors on the frequency of non-carious cervical lesions. Quantity and quality of saliva, as one of the potential etiological factor, was also evaluated.

## Material and Methods

-Subjects

Investigations were conducted on a sample of patients selected by convenience sampling method, who referred for dental examination and treatment, at the Department of Restorative dentistry and Endodontics, Faculty of Dentistry in Pancevo, Serbia. The study involved patients of both genders, aged over 18 years. The condition for participating in the study was to have a minimum of eight eligible teeth and to be able to read and understand the questionnaire that was used in this study. Patients with large quantities of calculus on teeth and patients that were currently wearing fixed orthodontic appliances were excluded. A sample size was originally calculated according to the “G*Power 3.1”, statistical power analysis program (3). The calculation was based on the analysis on a smaller preliminary sample and findings that the proportion of the respondents without lesions or up to 3 lesions and those with multiple NCCLs (more than 3) was about 60% versus 40%, and the odds ratio values for most of the tested variables were at least 1.5 or higher. Alpha was set to 5% and the power of 0.80 was considered acceptable. According to these parameters, a sample size of at least 313 participants would be required. The final study sample included 394 subjects (above the required number of 313), 169 male and 225 female patients, aged over 18 years. The youngest participant was 19 years old, while the oldest one was 81 years. All subjects were divided into three age groups: the first group consisted of subjects aged 19-35 years, the second group of subjects 36 to 55 years, and the third group of subjects over 55 years. A total number of 9499 teeth was examined. Before examination, the patients were fully informed about the study and each of them gave written consent to participate as a volunteer. All investigations in this study, i.e. the complete medical history, dental examination of patients, as well as taking the necessary samples, were conducted after approval by the “Ethics Committee for Research, Faculty of Dentistry in Pancevo” (Approval Protocol No. 882/1-2014, according to Resolution sections 3, 7, and 8 of the National Commission of Ethics in Research). The study procedures were conducted in complete accordance with the World Medical Association`s Declaration of Helsinki.

-Questionnaire

For the purpose of this study, a special questionnaire was developed, similar with those used in previously conducted epidemiological studies. It included questions about possible intrinsic and extrinsic factors of importance for the development of non-carious cervical lesions. On the first page of the questionnaire, basic information for each participant was recorded (first name, last name, gender, year of birth, place of residence, occupation, phone number). Patients were then asked about potential etiological factors related to general health and possible medication, dietary habits (consumption of citrus fruits and fruit drinks, carbonated and energy drinks), oral health behavior, lifestyle and some bad habits (smoking tobacco, narcotics, nail biting), presence of bruxism. Reliability of the questionnaire was tested using a test-retest correlation on a smaller preliminary sample of respondents at two distinct time periods. Correlation coefficient (r) was 0.86, which is considered good.

-Clinical examination 

Comfortable accommodation was provided in the dental chair, with adequate lighting. The patients were clinically examined by a standardised procedure for dental examination, using a dental mirror and a straight dental probe. Complete clinical examination for this study was carried out by one examiner, previously trained by written instructions, all in order to get more precise data, and avoid potential differences in diagnostic criteria. Intra-examiner agreement was calculated using Kappa statistics resulting from examination of 30 individuals with and without NCCLs at two distinct time periods with an interval of two weeks, following recommendations from WHO for reliability and validity of data. Cohen`s Kappa value index was 0.92, which is considered excellent.

-Quantitative and qualitative analysis of saliva 

Saliva samples were analyzed for a total of 60 patients. Samples were taken from 30 patients of both genders, who had been diagnosed multiple NCCLs (>3) and 30 patients of both genders, without any signs of cervical lesions and cervical restorations, who represented the control group. One day before collecting samples, patients were instructed via telephone interview not to eat, drink, smoke and brush their teeth for at least 60 minutes before arrival. Sampling procedure was carried out as recommended by “University of Southern California School of Dentistry guidelines for saliva collection” (4). The quantity of saliva was investigated by measuring the flow rate of unstimulated and stimulated saliva. Qualitative analysis included the determination of saliva acidity coefficient (pH) of stimulated and unstimulated saliva. Samples of unstimulated and stimulated saliva were collected in graduated containers, which were previously marked using first and last name of the patient and the number of their dental record. Determination of saliva samples acidity was done using pH-meter (830 C - Multi Parameter Analyzer, Consort and Turnhout, Belgium).

-Statistical analysis

Descriptive statistics was obtained using SAS statistical package (SAS Institute, 2010) for all the characteristics. Results were expressed as percentages, with mean values, standard deviation and minimum and maximum values for the respective groups of samples (gender, age). Subject - level analysis was used to analyze the association of possible etiological factors and presence of NCCLs. Considering potential different etiology of NCCLs and in order to obtain a more objective result, all subjects were divided into two groups. The first group included subjects without NCCLs and those with up to 3 NCCLs, while the second group consisted of subjects with multiple NCCLs (more than 3). Each factor was first employed as an independent variable in a univariate unconditional logistic regression, in which the dependent variable was the presence of multiple NCCLs. After that, the factors that showed a significant correlation with NCCLs in the univariate unconditional logistic analysis were used as independent variables in multivariate logistic analysis. Odds-ratio (OR) was used to show the strenght of association at significance level of P≤0.05 and 95% Confidence interval (CI) was also calculated. The logistic regression model was reviewed for goodness-of-fit and validated using the Hosmer - Lemeshow statistics. Wilcoxon test (SAS Institute, 2010) was used to analyze the quantity and pH value of unstimulated and stimulated saliva.

## Results

**Figure 1:**
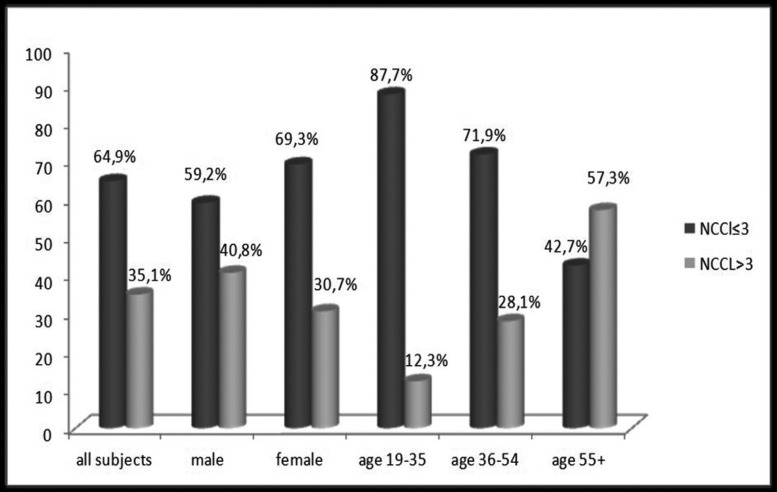
Distribution of subjects with multiple NCCL by gender and age groups.

The presence of NCCLs was diagnosed in 68.5% of total respondents. Among male respondents, the percentage of those with NCCLs was 76.3% and among female respondents 62.7%. The highest frequency of patients with NCCLs was recorded in age group older than 55 years (94.7%), while NCCLs were the least represented in the youngest age group (35.2%) ( Table 1). In more than one-third subjects of this study (35.1%) multiple NCCLs (>3) were diagnosed. Higher percentage of multiple NCCLs was recorded among male subjects. Percentage of patients with multiple lesions increases with age, with more than a half of them (57.3%) in group over 55 years old (Fig. 1).

**Table 1:**
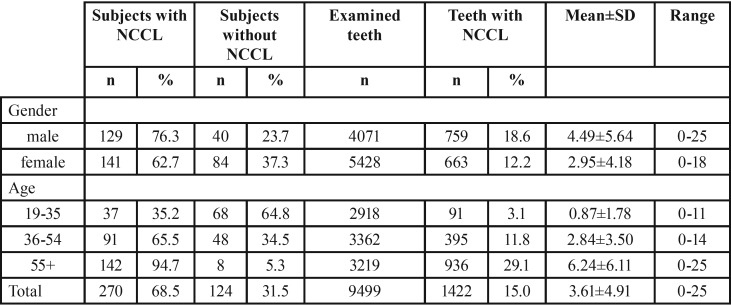
Distribution of subjects and teeth with NCCL by gender and age groups.

**Figure 2:**
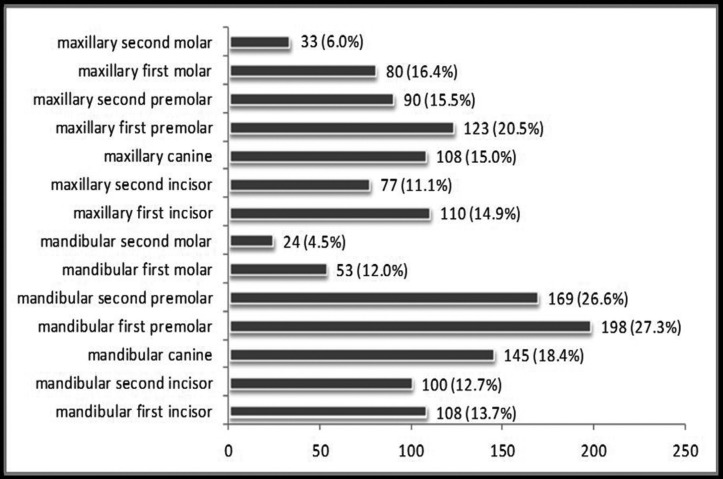
Number of teeth with NCCL.

From total number of 9499 teeth examined, 15% of them were affected by NCCLs. Higher percentage of teeth with NCCL was observed among male subjects (18.6%). The lowest percentage of teeth with NCCL was in the group of patients aged 19 to 35 years (3.1%), while the highest was in the group of patients older than 55 years (29.1%) ( Table 1). Among teeth functional groups, the highest frequency of NCCLs was recorded on premolars (22.8% from total number of premolars examined). Slightly higher percentage of lesions was recorded in the lower jaw (17.0% versus 14.2%) and on teeth of the left half of the dental arch (16.5% versus 14.8%). A far greater number of NCCLs was found on the facial surface compared to oral ( Table 2). Mandibular first premolars were the most frequently affected teeth (198, or 27.3% from total number of lower first premolars examined) (Fig. 2).

**Table 2:**
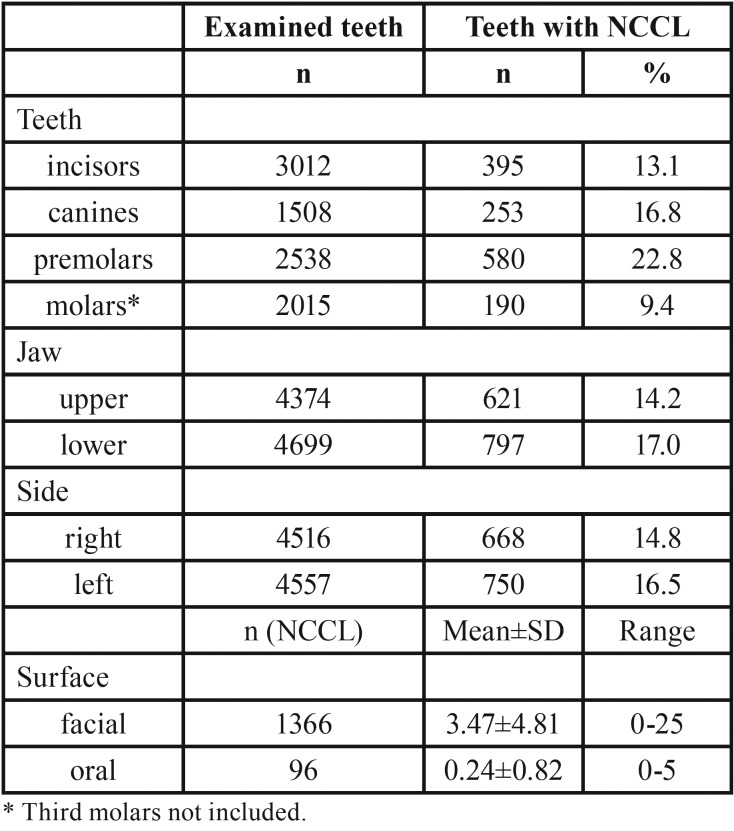
Distribution of NCCL by teeth functional group, jaw, arch side and tooth surface.

The results of univariate unconditional logistic regression showed a direct link between the presence of multiple NCCLs and gender (*p*=0.037), age (*p*<0.001) and frequent consumption of citrus fruits (p<0.001). Also, results of this study showed significantly lower frequency of NCCLs among those subjects who frequently chew gums (*p*<0.001). When it comes to the influence of oral hygiene factors, the analysis showed a significantly higher frequency of multiple lesions among subjects who brush their teeth once a day compared with those who brush 2 times or more (*p*=0.005) and among patients who doesn`t know the type of toothbrush they use (*p*=0.006). The multivariate logistic regression analysis revealed that only age (*p*<0.001), frequent consumption of citrus fruits (*p*=0.003) and chewing gums habit (*p*=0.001) were associated with the presence of multiple NCCLs ( Table 3).

**Table 3:**
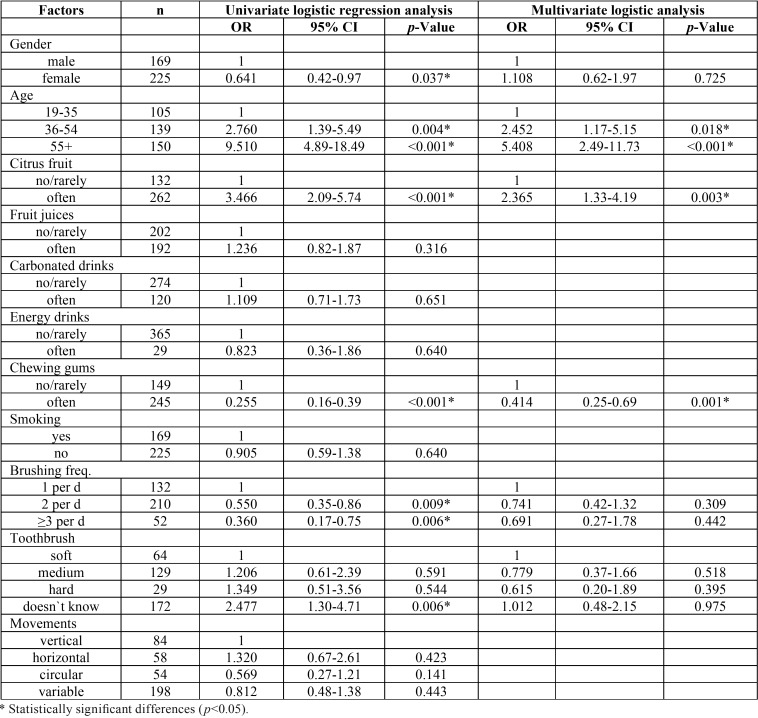
Results of univariate and multivariate logistic regression for the presence of NCCL.

Wilcoxon test showed no significant difference in terms of unstimulated and stimulated saliva flow rate among two groups of patients. On the other hand, significantly lower pH values of unstimulated (*p*=0.002) and stimulated saliva (*p*=0.004) were recorded in the group of patients with multiple NCCLs in comparison to control group ( Table 4).

**Table 4:**
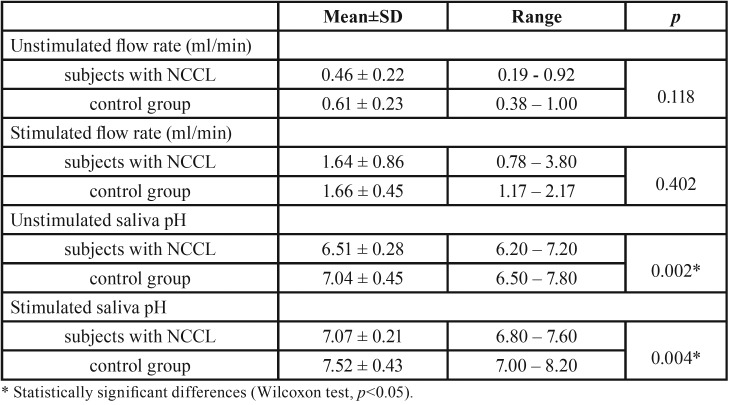
Unstimulated and stimulated saliva flow rate and salivary pH analysis (Wilcoxon test).

## Discussion

Data from the literature regarding prevalence of NCCLs show a high degree of discrepancy and are largely determined by various criteria for the assessment of lesions and their morphology. Review papers suggest that the prevalence ranges from 5% to 85% (5). More recent studies also show large differences regarding prevalence of NCCLs, ranging from 9% (6), 35% (7) up to 77% (8). The reasons for such results disparity can be found in a variety of test patterns (number of participants, age), different methodology, as well as differences in diagnostic criteria. This high variability may point the fact that it is very difficult precisely to define what actually presents a NCCL. A total of 68.5% subjects of this study had one or more teeth with NCCL. It is particularly interesting that very similar prevalence was observed in Borcic *et al.* study (9). The study was conducted on 1002 subjects from Rijeka (Croatia) and recorded prevalence of NCCLs was 65% respectively and 16.6% of all examined teeth. The agreement of two studies is interesting because of relatively close geographical area and it leaves space for investigation on the impact of certain cultural characteristics, habits and customs on the frequency of NCCLs.

NCCLs were diagnosed on 15% from a total number of 9499 examined teeth. There is a wide range of different results in the literature. For example, the percentage varies from only 1% to 74% (10,11). The reasons for such different results could be found in differences in diagnostic criteria and method of classification, and it is clear that percentage changes depending on whether it is calculated in relation to all present teeth or only in relation to teeth with lesions. NCCLs were most frequently observed in premolars (22.8% from the total number of present premolars) and lower first premolars were the most frequently affected teeth (27.3%). Data from the literature suggest that NCCLs can occur on any tooth, but there is a strong tendency among results suggesting that premolars are most common among them, namely first premolars (9,12). Various authors tried to explain this phenomenon offering different theories, such as frequent presence of premature occlusal contacts on premolars, limited protective effect by saliva, prolonged and strong abrasive brushing effect because of central position in dental arch, notable difference in cortical bone thickness on the vestibular and oral side of the tooth, cervical stress because of buccal cusps inclination during lateral movements (13).

A significant majority of NCCLs were diagnosed on the facial surface of the tooth (93% from total number of lesions). It should be noted that there was a certain number of teeth with lesions on both, facial and oral surface. Mainly similar results were recorded in other studies (14). The reasons for this could be found primarily in the fact that facial surface is more accessible for brushing action compared to oral surface. The lingual surface of the lower teeth is protected by tongue and saliva from submandibular and sublingual salivary glands, and is thus less prone to erosion. Nowadays, well known is abfraction theory, described as the loss of tooth structure due to the concentration of forces, particularly tensile stress. Rees *et al.* tried to explain this phenomenon by interaction between occlusal loads and erosion, so-called stress-corrosion (15).

In the present study, despite of the higher percentage of males with multiple lesions, no significant difference in the prevalence of NCCLs was found in relation to gender. This result is in compliance with the results of numerous previous epidemiological studies (1,6,8).

Results of the present study indicate that percentage of subjects with multiple NCCLs increases with age. Such results are consistent with a large number of studies (1,6,8,9,12). The most likely reasons for such distribution of NCCLs is cumulative effect of large number of etiological factors over a long period of time, larger degree of gingival recession, a smaller number of present teeth and thus a higher occlusal load, loss of the protective mechanisms of the natural dentition, reduced quality and quantity of saliva, structural and microstructural changes in enamel and dentin that are related to the aging process.

Etiology of NCCLs is a matter of numerous controversies even today. It is believed that these lesions occur as a result of different mechanisms, acting solo or simultaneosly. These mechanisms include: friction (abrasion and attrition), corrosion (chemical or electrochemical degradation) and stress, resulting in compression, flexion and tension, which in turn lead to microfractures and abfraction (16). Results of the present study confirmed significant association between frequent consumption of citrus fruits and frequency of NCCLs. Cervical tooth surface is prone to erosion because it is very close to the gingival margin, and therefore less prone to self-cleaning, and acid from foods and beverages may exhibit its erosive effect on the tooth surface for a longer period. This result is in agreement with the result of Bartlett *et al.* study which included almost 3000 subjects from seven European countries (17). The erosive potential of cola-type soft drinks, fruit juices and energy drinks is well known. The assumption is that the erosive potential of these drinks comes from high concentration of processed carbohydrates, which stimulate higher level of acid production, and therefore the higher titratable acidity (18-20). Despite slightly greater frequency of NCCLs among subjects who frequently consume fruit juices, carbonated and energy drinks, significant association was not recorded in the present study. One of the reasons could be that the questionnaire did not include questions relating to the exact type of drinks, because there are some differences in terms of titratable acidity.

Higher percentage of subjects in this study with multiple NCCLs was recorded among those who do not chew gums. Since it is known that chewing gums increase secretion of saliva, and thus increase neutralisation of certain acidic ingredients with erosive potential, the result of the present study could be regarded as expected, but it certainly points to the need for further research in this field. On the other hand, if we take into account the activation of the masticatory muscles and a large number of opposing teeth contacts during chewing, there is some space for the assumption that this habit for a longer period of time could pose some risk for attrition type of tooth wear. Data from the literature considering chewing gum as a potential factor of importance for the frequency of NCCLs are insufficient. Bartlett *et al.* who examined NCCLs in adolescents did not recorded significant association between the habit of chewing gum and the degree of cervical tooth substance wear (17).

In some studies, habit of smoking cigarettes is claimed as a potential etiological factor for NCCLs, primarily due to its influence on gingival recession, thereby indirectly providing conditions for NCCLs development (21). Significant association between cigarettes smoking and the frequency of NCCLs was not recorded in this study. One reason for such result could certainly be the fact that the majority of smokers were registered among younger subjects. A negligible number of subjects of this study indicated presence of parafunctional habits, and that was the reason why this factor was excluded from the analysis.

Habits related to dental hygiene, such as daily frequency, brushing technique or hardness of the toothbrush are considered to be potential factor of importance in the process of dental abrasion (7,12). Although prevalence of NCCLs slightly increased with increasing of toothbrush hardness and among subjects using horizontal brushing movements, no significant association between oral hygiene factors and prevalence of NCCLs was recorded in the present study after multivariate logistic regression analysis, probably because of the mutual dependence between these factors. Another reason for such a result might be due to the fact that only 7% of respondents in the present study used a hard toothbrush and majority of them (45%) didn`t even know to answer which type of toothbrush they use. These results indicate that the role of dental hygiene factors in the development of cervical lesions could only be considered as a potential alternative and complementary mechanism. Results of this study are in agreement with the results of Ibrahim *et al.*, Jafari and Bartlett *et al.* studies (6,8,17).

Saliva is considered as a biological modifying factor that can have an impact on the process of the development and progression of erosive lesions. According to some authors, the main factor of its protective role is contribution of saliva in creating pellicle. Acquired pellicle acts as a barrier which prevents contact between the dental tissue and the acid, all that in order to delay the erosive effect on tooth enamel. The protective role of saliva is also reflected in dilution and removal of erosive substances, in neutralization and acid buffering, in maintaining the supersaturated condition within the local environment of the tooth surface due to the presence of calcium and phosphate in saliva, in the fact that saliva presents reservoir of calcium, phosphate and possibly fluoride which are necessary for remineralisation (22). Different results can be found in various studies. While some authors reported significant association between reduced salivary flow rate and increased prevalence of dental erosion (23,24), others did not found that connection (25). When it comes to association between buffering capacity of saliva and NCCLs, according to the literature, results are also unconvincing. In some studies was recorded that the lower saliva buffer capacity was associated with an increased hard dental tisssue wear (26), and there are also authors who did not found this association (23,25). Unstimulated and stimulated saliva flow rate analysis in the present study showed slightly lower values among subjects with multiple NCCLs, but without statistical significance. On the other hand, pH values of unstimulated and stimulated saliva were significantly lower among subjects with multiple NCCLs, which leaves space for assumption that salivary pH value could be a factor of importance for initiation and progression of NCCLs. Results of this study are fully in agreement with the results of Dukić *et al.* who concluded that the most important factor for higher prevalence of dental erosions was lower pH value of stimulated and unstimulated saliva (27).

The limitations of the present study should be highlighted and considered. The study sample is not nationally representative and could be considered as a convenience sample. Although the patients attending the Faculty of Dentistry in Pancevo provide an easily accessible study sample of the Southern Banat area adult population, a study with a nationwide representative sample is needed. In the present study, cervical lesions have not been considered specifically by type (erosion, abrasion, abfraction), but cumulatively, so the results must be taken with caution. Occlusal factors were not included in the research. The data for the prevalence of NCCLs is descriptive, as it related to a clinical examination performed on the teeth. The analysis of the potential etiological factors is more subjective. The largest number of epidemiological studies dealing with the association between potential etiological factors and frequency of NCCLs relies on data obtained from specially designed questionnaires. Since a cross-sectional study addresses present time only, there is a possibility that some habits have changed, so questionnaire based data are sometimes not reliable enough. Correlation between different variables in one group of individuals might appear just by coincidence, so a new sample population is necessary to get more concrete results. Since this was a cross-sectional study, the causal relationship between some etiological factors and prevalence of NCCLs remains unclear and longitudinal studies are needed to confirm these results.

In conclusion, the results of the present study indicated relatively high frequency of non-carious cervical lesions among subjects of different age. Premolars were the most frequently affected teeth. Age, frequent consumption of citrus fruit and lower salivary pH value were associated with an increased occurrence of NCCLs, while chewing gums habit was associated with an decreased occurrence of NCCLs. Undoubtedly, neither solo etiological factor can be certainly considered as a factor that leads to NCCL development and progression, but can be considered as an individual dominant factor. Comprehensive results of this study might suggest multifactorial nature of NCCLs. Determination and early elimination of dominant etiological factor could be the first strategy step to prevent this common dental condition.
